# Supplementary material for: Zebrafish Embryonic Exposure to BPAP and Its Relatively Weak Thyroid Hormone-Disrupting Effects
Source: Toxics. 2020 Nov 13;8(4):103. doi: 10.3390/toxics8040103 (PMC7712898; doi:10.3390/toxics8040103)
Supplement: Supplementary file 1 [file toxics-08-00103-s001.pdf]

Supplementary Materials: Zebrafish Embryonic Exposure to BPAP and Its Relatively Weak Thyroid Hormone-Disrupting Effects

Sangwoo Lee, Kojo Eghan, Jieon Lee, Donggon Yoo, Seokjoo Yoon and Woo-Keun Kim

1. Methods and Materials

1.1. RNA Sequencing

RNA samples from the control group and 105.9 μg/L BPAP-exposed group were prepared and maintained in RNAlater® RNA stabilization solution (Qiagen, Hilden, Germany) for further RNA extraction. Total RNA was extracted using the RNeasy mini kit (Qiagen, Hilden, Germany). The tRNA band integrity was assessed using an Agilent RNA 6000 Nano kit (Agilent Technologies, Palo Alto, CA, USA). Samples with an RNA integrity number (RIN) greater than 7 were used for RNA library construction. Briefly, prior to cDNA library construction, 1 μg of total RNA and magnetic beads with oligo (dT) were used for poly (A) mRNA enrichment. The purified mRNA was then disrupted into short fragments, and double-stranded cDNA was immediately synthesized. The cDNA was subjected to end-repair and poly (A) addition and connected with sequencing adapters using the TruSeq RNA Sample Prep Kit (Illumina Inc., San Diego, CA, USA). The suitable fragments, which were purified with a BluePippin 2% agarose gel cassette (Sage Science, Beverley, MA, USA), were selected as templates for PCR amplification. The final libraries were quantified using a KAPA library quantification kit (KAPA Biosystems, Wilmington, MA, USA), and the quality of the library was evaluated using an Agilent 2100 Bioanalyzer (Agilent Technologies, Palo Alto, CA, USA). The fragments contained between 350 and 450 base pairs. The library was then sequenced using an Illumina HiSeq2500 sequencing platform (Illumina Inc., San Diego, CA, USA). Low-quality reads were filtered out according to the following criteria: reads containing more than 10% skipped bases, reads containing more than 40% of bases whose quality scores were less than 20, and reads with average quality scores less than 20. The filtered reads were mapped to the zebrafish reference genome (Ensembl version 86) using the aligner STAR v.2.3.0e

1.2. DEG Analysis

We measured the gene expression levels using Cufflinks v2.1.1 [1] and the gene annotation database from Ensembl version 86. The noncoding gene region was removed with the mask option. To improve the accuracy of the measurements, we applied multiread correction and fragment bias correction. The abundance of gene transcripts was measured by FPKM (fragments per kilobase of transcript per million fragments mapped). The FPKM cut-off level was set to 0.1. For differential expression analysis, gene level count data were generated using the HTSeq-count v0.5.4p3 tool [2]. Using the calculated read count data, differentially expressed genes (DEGs) were identified using the TCC R package [3].

Table S1. Molecular structure and characteristics of BPAP.

Chemicals	Structure	CAS RN.	Purity (%)	MW (g/mol)	LogK$_{OW}$

4,4′-(1-Phenylethylidene) bisphenol	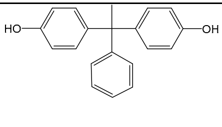	1571-75-1	99	290.36	4.86

Table S2. Primer sequences used in the qRT-PCR analysis in the present study.

Gene	Primer Sequence (5′–3′)		Accession No.
	Forward	Reverse	
18srRNA[a]	acgcgagatggagcaataac	cctcgttgatgggaaacagt	FJ915075
crh[b]	ttcgggaagtaaccacaagc	ctgcactctattcgccttcc	NM_001007379.1
tshβ[c]	gcagatcctcacttcacctacc	gcacaggtttggagcatctca	AY135147
nkx2.1[c]	aggacggtaaaccgtgtcag	caccatgctgctcgtgtact	BC162296.1
hhex	tgtggtctccgttcatccag	tttgacctgtctctcgctga	NM_130934
tshr	gcgccaaccctttctgtat	ctcgtttgctcctgtttgct	EF487539.1
slc5a5	ggtggcatgaaggctgtaat	gatacggcatccattgttgg	NM_001089391
tg[c]	ccagccgaaaggatagagttg	atgctgccgtggaatagga	XM 001335283
pax8[c]	gaagatcgcggagtacaagc	ctgcactttagtgcggatga	AF072549
tpo[b]	gcgcttggaacacagtatca	cttcagcaccaaacccaaat	EU267076
trα[a]	caatgtaccatttcgcgttg	gctcctgctctgtgttttcc	NM_131396
trβ[b]	tgggagatgatacgggttgt	ataggtgccgatccaatgtc	NM_131340
ttr[c]	cgggtggagtttgacacttt	gctcagaaggagagccagta	BC081488
dio1[c]	gttcaaacagcttgtcaaggact	agcaagcctctcctccaagtt	BC076008
dio2	ttctccttgcctcctcagtg	agccacctccgaacatcttt	NM_212789.3
dio3	gagaccgctgatcctcaacttc	tcgatgtacaccagcagagagt	NM_001177935.3
ugt1ab[c]	ccaccaagtctttccgtgtt	gcagtccttcacaggctttc	NM_213422

[a] Liu et al. (2013) [4]; [b] Yu et al. (2010) [5]; [c] Wang et al. (2013)[6]; primers for which a reference is not denoted were designed using Primer 3 online software ver. 4.0.0 (http://primer3.ut.ee/).

Table S3. Ingenuity canonical pathways determined by IPA.

Ingenuity Canonical Pathways	−log(*p*-value)	Ratio
Production of Nitric Oxide and Reactive Oxygen Species in Macrophages	5.16	0.0408
Endothelin-1 Signaling	5.11	0.0402
IL-12 Signaling and Production in Macrophages	4.99	0.0473
Neuroinflammation Signaling Pathway	4.52	0.0288
Interferon Signaling	4.5	0.111
Huntington's Disease Signaling	4.38	0.0317
Atherosclerosis Signaling	4.33	0.0469
LXR/RXR Activation	3.43	0.0413
HIF1α Signaling	3.35	0.0397
Airway Pathology in Chronic Obstructive Pulmonary Disease	3.16	0.25
Antigen Presentation Pathway	3.03	0.0789
Inhibition of Matrix Metalloproteases	3	0.0769
MIF Regulation of Innate Immunity	2.91	0.0714
Role of RIG1-like Receptors in Antiviral Innate Immunity	2.85	0.0682
Relaxin Signaling	2.85	0.0307
Synaptic Long Term Depression	2.6	0.0269

FXR/RXR Activation	2.42	0.0317
Osteoarthritis Pathway	2.36	0.0237
Leukocyte Extravasation Signaling	2.35	0.0235
Maturity Onset Diabetes of Young (MODY) Signaling	2.3	0.0952
Polyamine Regulation in Colon Cancer	2.26	0.0909
Pyrimidine Deoxyribonucleotides De Novo Biosynthesis I	2.23	0.087
Endocannabinoid Cancer Inhibition Pathway	2.07	0.0253
Colorectal Cancer Metastasis Signaling	2.02	0.0196
UDP-D-xylose and UDP-D-glucuronate Biosynthesis	2.00	0.5
Flavin Biosynthesis IV (Mammalian)	2.00	0.5
Prolactin Signaling	1.96	0.033
Glutathione-mediated Detoxification	1.95	0.0625
Bladder Cancer Signaling	1.92	0.0319
MIF-mediated Glucocorticoid Regulation	1.9	0.0588
VEGF Family Ligand-Receptor Interactions	1.9	0.0312
PPARα/RXRα Activation	1.84	0.0215
Complement System	1.83	0.0541
D-glucuronate Degradation I	1.82	0.333
Inosine-5′-phosphate Biosynthesis II	1.82	0.333
L-serine Degradation	1.82	0.333
Antioxidant Action of Vitamin C	1.75	0.0275
Type I Diabetes Mellitus Signaling	1.73	0.027
Amyotrophic Lateral Sclerosis Signaling	1.71	0.0265
IGF-1 Signaling	1.71	0.0265
Arsenate Detoxification I (Glutaredoxin)	1.70	0.25
Ascorbate Recycling (Cytosolic)	1.70	0.25
Clathrin-mediated Endocytosis Signaling	1.67	0.0191
iNOS Signaling	1.67	0.0444
Trehalose Degradation II (Trehalase)	1.60	0.2
Tetrahydrofolate Salvage from 5,10-Methenyltetrahydrofolate	1.60	0.2
Citrulline-Nitric Oxide Cycle	1.60	0.2
Folate Polyglutamylation	1.60	0.2
Sphingosine-1-phosphate Signaling	1.59	0.0238
Fc Epsilon RI Signaling	1.58	0.0236
Role of Tissue Factor in Cancer	1.53	0.0227
CCR3 Signaling in Eosinophils	1.49	0.0219
Th1 Pathway	1.49	0.0219
Role of Pattern Recognition Receptors in Recognition of Bacteria and Viruses	1.48	0.0216
Adenine and Adenosine Salvage III	1.46	0.143
Signaling by Rho Family GTPases	1.41	0.0159
Phagosome Maturation	1.41	0.0203
Retinoic Acid-mediated Apoptosis Signaling	1.41	0.0323
Histidine Degradation III	1.40	0.125
Purine Ribonucleosides Degradation to Ribose-1-phosphate	1.40	0.125
Axonal Guidance Signaling	1.39	0.012

Activation of IRF by Cytosolic Pattern-Recognition Receptors	1.39	0.0317
Phospholipases	1.39	0.0317
Type II Diabetes Mellitus Signaling	1.36	0.0194
Folate Transformations I	1.35	0.111
Eicosanoid Signaling	1.34	0.0294
Protein Ubiquitination Pathway	1.32	0.0148
Role of IL-17A in Arthritis	1.31	0.0286
Gαq Signaling	1.31	0.0185
GDP-glucose Biosynthesis	1.31	0.1
Glycine Betaine Degradation	1.31	0.1
Ephrin B Signaling	1.29	0.0278
IL-2 Signaling	1.29	0.0278
GPCR-Mediated Integration of Enteroendocrine Signaling Exemplified by an L Cell	1.28	0.0274
MSP-RON Signaling Pathway	1.27	0.027
Glucose and Glucose-1-phosphate Degradation	1.27	0.0909
Purine Nucleotides De Novo Biosynthesis II	1.27	0.0909
UDP-N-acetyl-D-galactosamine Biosynthesis II	1.27	0.0909
TREM1 Signaling	1.26	0.0267
Tec Kinase Signaling	1.26	0.0175
CXCR4 Signaling	1.25	0.0174
Heparan Sulfate Biosynthesis (Late Stages)	1.25	0.0263
VDR/RXR Activation	1.23	0.0256
Glioma Invasiveness Signaling	1.23	0.0256
Role of JAK1 and JAK3 in γc Cytokine Signaling	1.23	0.0256
Acute Phase Response Signaling	1.23	0.017
Role of MAPK Signaling in the Pathogenesis of Influenza	1.22	0.0253
IL-17A Signaling in Airway Cells	1.21	0.025
Granulocyte Adhesion and Diapedesis	1.20	0.0167
Oleate Biosynthesis II (Animals)	1.20	0.0769
γ-Glutamyl Cycle	1.20	0.0769
Xenobiotic Metabolism Signaling	1.19	0.0134
Heparan Sulfate Biosynthesis	1.18	0.0241
Colanic Acid Building Blocks Biosynthesis	1.17	0.0714
Th1 and Th2 Activation Pathway	1.16	0.016
Growth Hormone Signaling	1.15	0.0233
Erythropoietin Signaling	1.14	0.0227
Superpathway of Citrulline Metabolism	1.14	0.0667
Agranulocyte Adhesion and Diapedesis	1.14	0.0156
Role of NFAT in Regulation of the Immune Response	1.13	0.0155
Dendritic Cell Maturation	1.12	0.0153
IL-1 Signaling	1.11	0.022
JAK/Stat Signaling	1.11	0.022
ILK Signaling	1.11	0.0152
Adenosine Nucleotides Degradation II	1.11	0.0625
Vitamin-C Transport	1.11	0.0625

IL-17 Signaling	1.10	0.0215
Death Receptor Signaling	1.10	0.0215
IL-7 Signaling Pathway	1.10	0.0215
NRF2-mediated Oxidative Stress Response	1.08	0.0149
γ-Linolenate Biosynthesis II (Animals)	1.08	0.0588
IL-8 Signaling	1.07	0.0147
IL-4 Signaling	1.07	0.0208
Apoptosis Signaling	1.07	0.0208
ERK/MAPK Signaling	1.07	0.0146
Adrenomedullin Signaling Pathway	1.06	0.0145
Thrombin Signaling	1.04	0.0142
Purine Nucleotides Degradation II (Aerobic)	1.04	0.0526
CTLA4 Signaling in Cytotoxic T Lymphocytes	1.04	0.0198
Visual Cycle	1.02	0.05
Inflammasome pathway	1.02	0.05
CREB Signaling in Neurons	1.00	0.0137

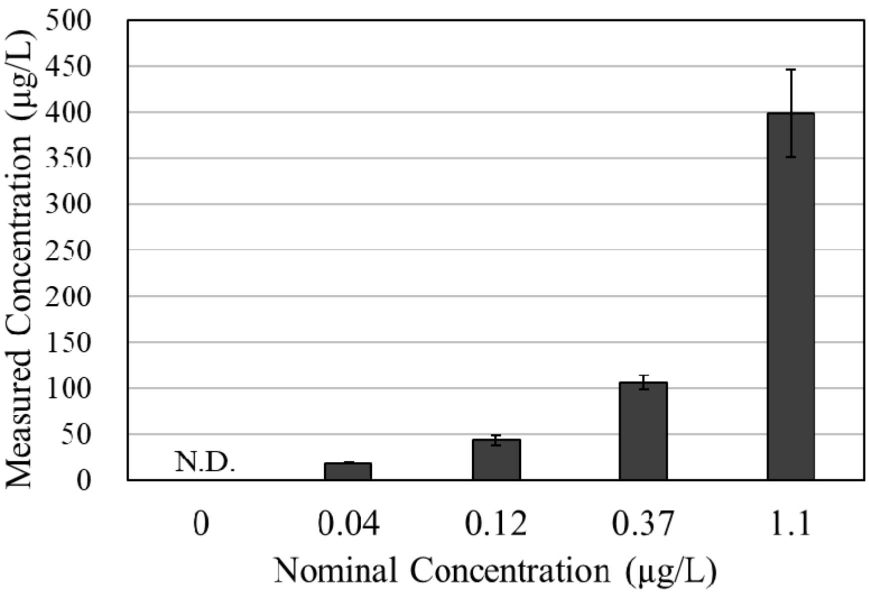

Figure S1. Measured concentrations of BPAP. The LOQ for BPAP in calibration curve was 8.5 ng/mL in this study. N.D.: BPAP was not detected in the SC group.

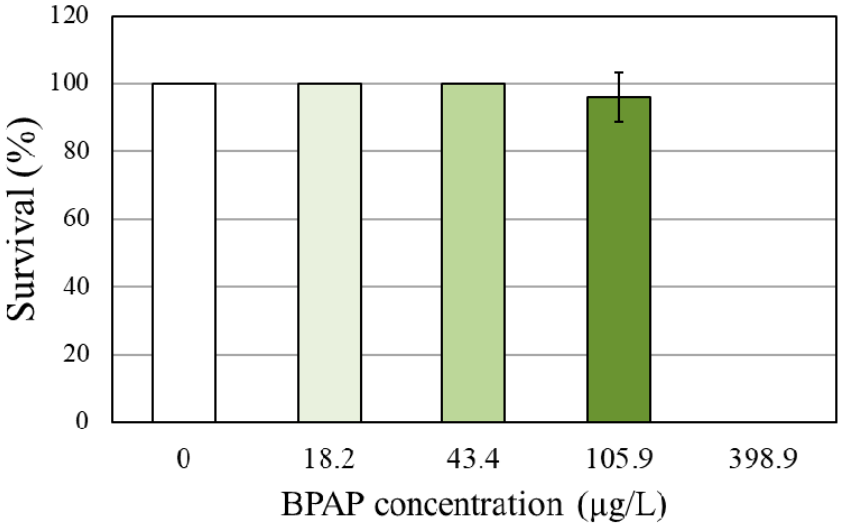

Figure S2. Survival of zebrafish embryos/larvae after 120 h of exposure to BPAP. The results are shown as the means ± SDs (N = 3). All zebrafish larvae died after exposure to 398.9 μg/L BPAP.

(a)

(b
)

(c)

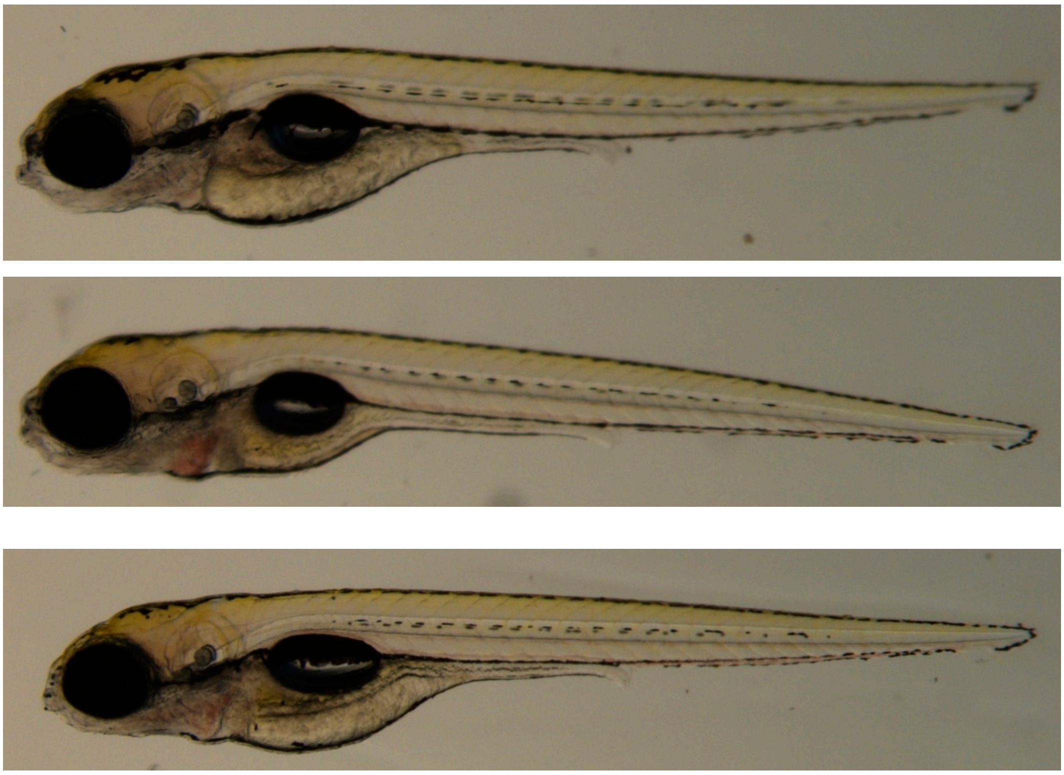

(d
)

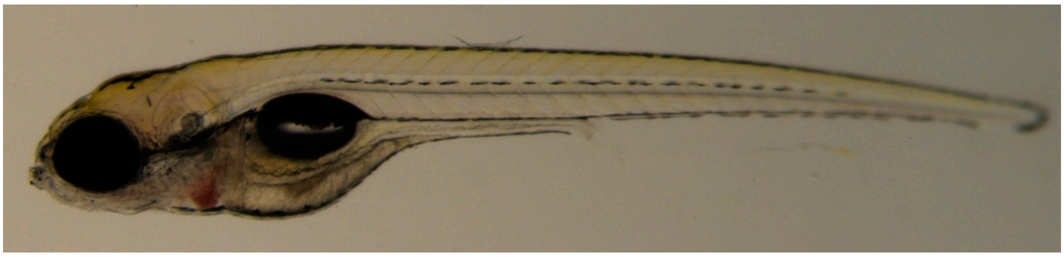

Figure S3. Morphology of zebrafish larvae after 120 h of exposure to (**a**) the solvent control (0.1% *v*/*v* DMSO) and (**b**) 18.2 μg/L, (**c**) 43.4 μg/L and (**d**) 105.9 μg/L BPAP.

(a)

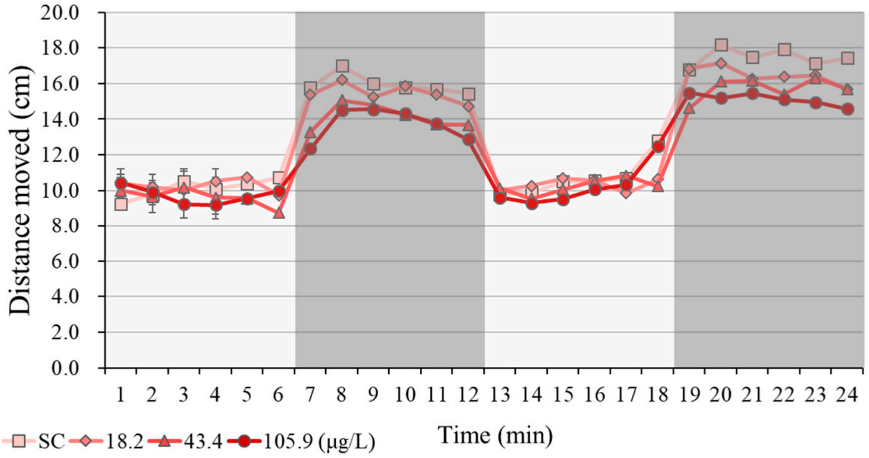

(b)

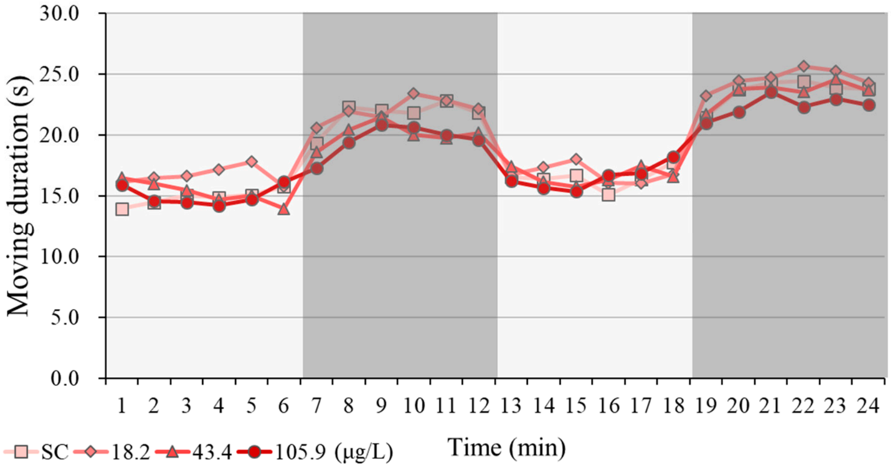

Figure S4. Locomotor activity ((**a**) distance moved and (**b**) moving duration)) per minute obtained for the zebrafish larvae after BPAP exposure (*N* = 6).

References

1. Trapnell, C.; Pachter, L.; Salzberg, S.L.; TopHat: discovering splice junctions with RNA-*Seq. Bioinform.* **2009,** *25*, 1105–1111 doi: 10.1093/bioinformatics/btp120.

2. Anders, S.; Pyl, P.T., Huber, W. HTSeq—a Python framework to work with high-throughput sequencing data. *Bioinform.* **2015,** *31*, 166–169 doi: 10.1093/bioinformatics/btu638.
3. Sun, J.; Nishiyama, T.; Shimizu, K.; Kadota, K. TCC: an R package for comparing tag count data with robust normalization strategies. *BMC Bioinform.* **2013**, *14*, 219. doi: 10.1186/1471-2105-14-219.
4. Liu, C.; Wang, Q.; Liang, K.; Liu, J.; Zhou, B.; Zhang, X.; Liu, H.; Giesy, J.P.; Yu, H. Effects of tris (1, 3-dichloro-2-propyl) phosphate and triphenyl phosphate on receptor-associated mRNA expression in zebrafish embryos/larvae. *Aquat. Toxicol.* **2013**, *128*, 147–157, 10.1016/j.aquatox.2012.12.010
5. Yu, L.; Deng, J.; Shi, X.; Liu, C.; Yu, K.; Zhou, B. Exposure to DE-71 alters thyroid hormone levels and gene transcription in the hypothalamic-pituitary-thyroid axis of zebrafish larvae. *Aquat. Toxicol.* **2010**, *97*, 226–233, 10.1016/j.aquatox.2009.10.022
6. Wang, Q.; Liang, K.; Liu, J.; Yang, L.; Guo, Y.; Liu, C.; Zhou, B. Exposure of zebrafish embryos/larvae to TDCPP alters concentrations of thyroid hormones and transcriptions of genes involved in the hypothalamic-pituitary-thyroid axis. *Aquat. Toxicol.* **2013**, *126*, 207–213, 10.1016/j.aquatox.2012.11.009
